# SCYL1 does not regulate REST expression and turnover

**DOI:** 10.1371/journal.pone.0178680

**Published:** 2017-06-01

**Authors:** Sebastien Gingras, Emin Kuliyev, Stéphane Pelletier

**Affiliations:** 1 Department of Immunology, University of Pittsburgh School of Medicine, Pittsburgh, Pennsylvania, United States of America; 2 Embryonic Stem Cell Laboratory, Department of Immunology, St. Jude Children’s Research Hospital, Memphis, Tennessee, United States of America; Rajiv Gandhi Centre for Biotechnology, INDIA

## Abstract

A recent study identified SCYL1 as one of the components of the oncogenic STP axis, which promotes triple-negative breast cancer by regulating degradation of the REST tumor suppressor. Contrary to the findings of that study, herein we show by using 3 distinct genetic approaches that SCYL1 does not regulate REST turnover. Specifically, REST protein levels and turnover were identical in *Scyl1+/+* and *Scyl1-/-* mouse embryonic fibroblasts. Similarly, targeted inactivation of *SCYL1* in Hek293T cells by using CRIPSR-Cas9 technology did not affect REST steady-state level and turnover. Furthermore, RNA interference–mediated depletion of SCYL1 in Hek293T or MDA-MB-231 cells did not alter REST steady-state level and turnover. Together, our findings indicate that SCYL1 does not contribute to REST turnover and thus do not support a previous study suggesting a role for SCYL1 in mediating REST degradation.

## Introduction

The RE-1 silencing transcription factor (REST), also known as the neuron-restrictive silencer factor, is a transcriptional repressor that regulates early lineage commitment and neuronal plasticity [1]. During the transition from pluripotent to neural stem/progenitor cells, REST is actively degraded by the proteasome [1, 2], and downregulation of REST triggers neuronal gene expression critical to nervous system development [1]. In addition to regulating neuronal development, REST also functions as a tumor suppressor in epithelial tissues. An RNA interference (RNAi)–based screen identified REST as an important regulator of human mammary epithelial cell proliferation [3]. Consistent with the role of REST in regulating epithelial cell transformation, inactivating mutations, gene deletions, gene silencing, and alternative splicing of *REST* have been associated with epithelial malignancies [3–6]. During neuronal development and epithelial cell transformation, REST is degraded by the ubiquitin-proteasome pathway through the recognition of a phospho-degron located in the C-terminal region by the ubiquitin ligase βTRCP [1, 2]. In search of signaling pathways that regulate the phosphorylation and degradation of REST, Karlin et al. identified a novel oncogenic complex comprising SCYL1, TEX14, and PLK1 (STP axis), which cooperatively promotes the degradation of REST by phosphorylating a conserved phospho-degron, thereby linking REST to βTRCP [7].

SCYL1 is a critical regulator of motor neuron survival [8–10]. In mice, loss of SCYL1 function leads to an early onset motor neuron disorder with characteristic features of amyotrophic lateral sclerosis (ALS), including mislocalization of TDP43 and ubiquilin-2 in cytoplasmic aggregates in spinal motor neurons [9]. The mechanism underlying the neuroprotective function of SCYL1, however, has remained elusive. Interestingly, an early study in chicken embryos showed that ectopic expression of REST in the developing spinal cord leads to axonal pathfinding errors and suggested that downregulation of REST is necessary for proper neuronal development [11]. In light of recent findings suggesting a role for SCYL1 in regulating REST [7] turnover, we hypothesized that REST could be stabilized in *Scyl1*-deficient mice causing errors in motor neuron pathfindings and motor neuron degeneration.

To test this hypothesis, we first validated the findings of Karlin et al. that suggest a role for SCYL1 in regulating REST expression and turnover by using 3 distinct genetic approaches. Unexpectedly, we found that SCYL1 is dispensable for REST turnover. Specifically, we show that targeted inactivation of *Scyl1* by conventional gene targeting in mouse embryonic fibroblasts (MEFs), CRIPSR-Cas9–mediated inactivation of *SCYL1* in Hek293T cells, or RNAi-mediated knockdown of *SCYL1* in Hek293T or MDA-MB-231 cells did not affect the steady-state expression level and turnover of REST. Thus, our data do not support the previously published data by Karlin et al. demonstrating a role for SCYL1 in regulating the REST level and turnover.

## Materials and methods

### Antibodies

The anti-REST rabbit polyclonal antibody (07–579) was purchased from EMD Millipore. The polyclonal anti-SCYL1 antibody produced in rabbit, Human Protein Atlas Number HPA015015, was purchased from Sigma-Aldrich. The monoclonal anti-β-actin antibody clone AC-15 produced in mouse (A1978) was purchased from Sigma-Aldrich. The anti-rabbit IgG HRP-linked antibody (111-035-003) and anti-mouse IgG HRP-linked antibody (115-035-174) were purchased from Jackson ImmunoResearch.

### Tissue culture

Hek293T cells (ATCC® CRL-3216™) and SV40-transformed MEFs obtained from E15 *Scyl1+/+* and *Scyl1-/-* mouse embryos [9] were cultured in DMEM, high glucose, supplemented with FBS, L-glutamine, penicillin, and streptomycin. MDA-MB-231 (ATCC® HTB-26™) cells were cultured in L-15 medium (Sigma-Aldrich, SLBN4238V), supplemented with fetal bovine serum to a final concentration of 10%, penicillin, and streptomycin. MDA-MB-231 cells were cultured with atmospheric air.

### Generation CRISPR-Cas9 constructs

*SCYL1* gene ablation in Hek293T cells was performed by using CRISPR-Cas9 technology. Two single-guide RNAs (sgRNAs) targeting exon 4 of *SCYL1* were selected as described previously [12]: hScyl1sgRNA1, TCGTGGACCGAGCTGGCGAG (chr11:65,526,191–65,526,213, GRCh38/hg38) and the hScyl1sgRNA2, CTGCTGTCAGCCAACTCCGG (chr11:65,526,304–65,526,326, GRCh38/hg38). The selected guide sequences have no potential off-target sites with fewer than 3 mismatches, and the protospacer-adjacent motif-proximal 13-nucleotide seed sequences are unique in the human genome. The guide sequences were cloned in PX458 to generate px458-hScyl1-1 and px458-hScyl1-2, respectively. pSpCas9(BB)-2A-GFP (PX458) was a gift from Dr. Feng Zhang (Addgene plasmid # 48138) [13].

### Generation of *SCYL1*-deficient Hek293T cells

Hek293T cells were transfected with both sgRNAs. Two days later, GFP-positive cells were single sorted by fluorescence-activated cell sorting into individual wells of 96-well plates. The region surrounding the target sequences was PCR amplified with the following primers: hScyl1-F01, GAGATCTCCTGGGGGCTACA, and hScyl1-R01, CAGAATCACCCAACCCCGAA. PCR products were TOPO cloned, and more than 12 mini-preps for each clone were sequenced. Three clones were obtained in which both *SCYL1* alleles contained distinct deletions that caused a frame shift and premature stop codon, leading to mRNA decay. Also, 3 additional clones were selected in which *SCYL1* remained unaltered. *SCYL1* deletion was confirmed by western blot analysis.

### Depletion of SCYL1 by RNA interference

Hek293T and MDA-MB-231 cells were transfected by using Lipofectamine® RNAiMAX transfection reagents (Invitrogen), as per manufacturer’s instructions, with the following RNAi duplexes (Invitrogen): SCYL1HSS126245 (5′-CCGUUGGGAAUAUACCUCAAGGCGA-3′), SCYL1HSS183826 (5′-CCAACCUCAAUGAGGAGCUGAUGAA-3′) and SCYL1HSS183827 (5′-UGCAACACCACAGUCUGCCUGGGCA-3′). Then, 30 pmole of RNAi duplexes in 250 μL Opti-MEM®I Reduced-Serum Medium without serum were combined with 5 μL Lipofectamine® RNAiMAX transfection reagents in 250 μL Opti-MEM®I Reduced Serum Medium without serum and mixed gently. After 20 min at room temperature, the RNAi duplex–lipofectamine complexes were added to each well of a 6-well plate containing 2.5 mL of culture medium. At 48 h post-transfection, cells were treated as described below.

### REST half-life

*Scyl1+/+* and *Scyl1-/-* MEFs, Hek293T cells, Hek293T-*SCYL1KO* cells and RNAi-treated Hek293T or MDA-MB-231 cells were plated in 6-well plates. At 24 (MEFs and Hek293T-SCYL1KO cells) or 48 h post-plating/transfection, cells were treated with 10 μg/mL of cycloheximide (CHX) for the indicated times. Cells were then lysed in RIPA buffer (50 mM Tris-HCl, pH 8.0, 150 mM NaCl, 1% NP-40 [Igepal-CA-630], 0.5% sodium deoxycholate, 0.1% SDS) containing Complete™ protease inhibitors and PhosSTOP™ phosphatase inhibitors (Roche). Endogenous REST, SCYL1, and β-actin protein levels were determined by western blot analysis, and band intensities were quantified by using NIH Image J software version 1.42q (http://rsbweb.nih.gov/ij).

### Cell cycle analysis by flow cytometry

Hek293T and Hek293T-SCYL1KO cells were incubated for 6 hours in the presence of DMSO or BI 2536 (100nM). The cells were then harvested and washed three times in PBS. The cells were then fixed in cold 70% ethanol for 30 minutes on ice. Next, the cells were washed twice with PBS, spun down and treated with 50 μL of RNase A (100 μg/mL). Then 200uL of propidium Iodide (50 μg/mL) was added to the cells and the cells were analyzed by flow cytometry (FACScan, Becton Dickinson, San Jose, CA). The % of cells in each stage of the cell cycle was determined using ModFit (Verity Software house, Topsham, ME) and plotted.

## Results

**Fig 1:**
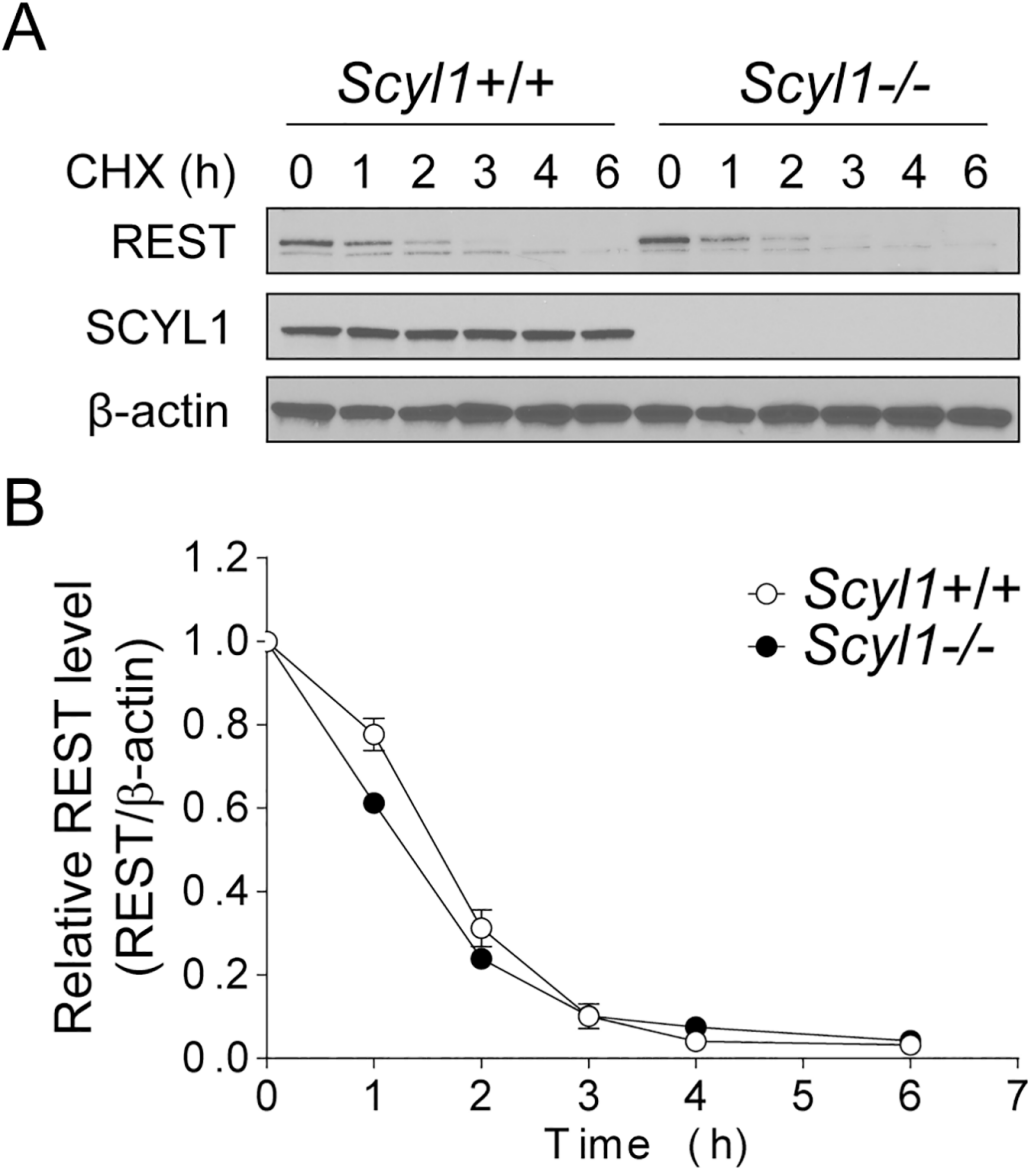
REST protein turnover and levels in *Scyl1+/+* and *Scyl1-/-* mouse embryonic fibroblasts (MEFs). **(**A) REST protein turnover in *Scyl1+/+* and *Scyl1-/-* MEFs. MEFs were seeded at a density of 250,000 cells per well of 6-well plates. The following day, cells were treated with 10 μg/mL of cycloheximide (CHX) for the indicated times. Endogenous REST, SCYL1, and β-actin levels were determined by western blot analysis. The images are representative of 2 independent experiments performed on 2 independently derived *Scyl1+/+* and 3 *Scyl1-/-* MEF lines. Note the absence of SCYL1 in *Scyl1-/-* MEFs. (B) Quantification of REST protein levels in *Scyl1+/+* and *Scyl1-/-* MEFs. The ratio of the band intensity of REST to β-actin was normalized to the ratio of untreated cells (time = 0 h). Data represent mean ± standard error of the mean.

To determine whether SCYL1 was involved in REST turnover, we measured the steady-state level and half-life of REST in *Scyl1+/+* and *Scyl1-/-* MEFs by using cycloheximide chase experiments. We recognize the fact that protein synthesis inhibition by CHX can alter protein degradation via AKT activation [14]and that 35S-methionine/cysteine metabolic labeling represents a better approach to follow the degradation of proteins. However, for the sake of reproducibility, we have used CHX to prevent de novo synthesis of REST as did Karlin et al. in their studies [7]. Unexpectedly, both the steady-state level and half-life of REST were identical in *Scyl1+/+* and *Scyl1-/-* MEFs (Fig 1), indicating that SCYL1 is dispensable for REST turnover in murine fibroblasts.

**Fig 2:**
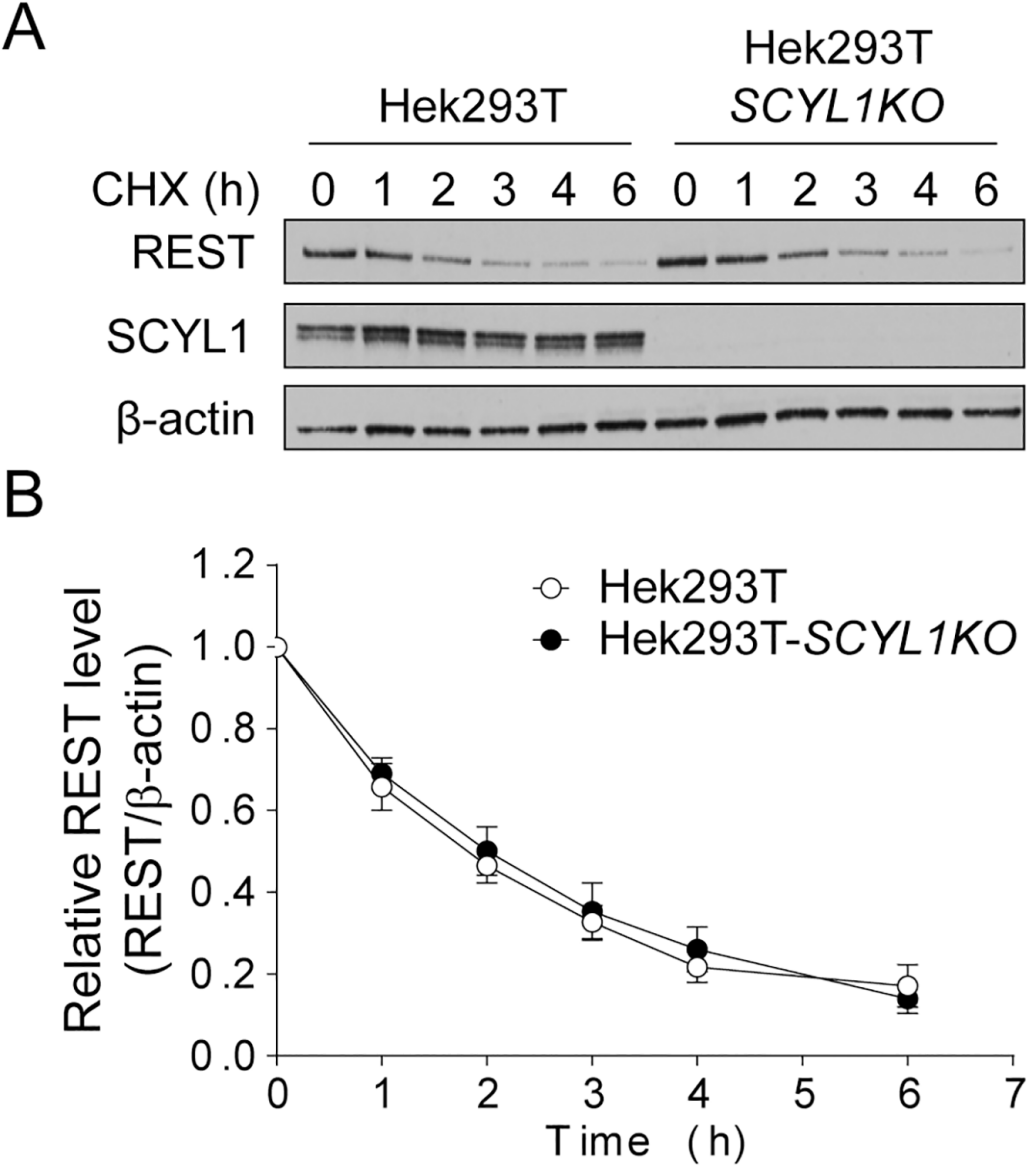
REST protein turnover and expression in Hek293T and Hek293T-*SCYL1*KO cells. (A) REST protein turnover in Hek293T cells. Hek293T-SCYL1KO cell lines (n = 3) were generated by using CRISPR-Cas9 technology (see Materials and Methods). Three Hek293T cell lines in which *SCYL1* was unaltered (Hek293T) and three Hek293T-SCYL1KO cell lines were seeded at a density of 250,000 cells per well of 6-well plates. The following day, the cells were treated with 10 μg/mL of CHX for the indicated time. Endogenous REST, SCYL1, and β-actin levels were determined by western blot analysis. The images are representative of 2 independent experiments performed on 3 different independently derived clones for each genotype. (B) Quantification of REST protein levels in cell lines Hek293T and Hek293T-*SCYL1KO*. The ratio of the band intensity of REST to β-actin was normalized to the ratio of untreated (time = 0 h). Data represent mean ± standard error of the mean.

Because MEFs may express factors that regulate REST turnover independently of the STP axis, we tested whether targeted ablation of *SCYL1* in Hek293T cells, the cells used in the Karlin et al. study, affected REST turnover. We generated 3 independent Hek293T cell lines in which *SCYL1* was disrupted by using CRISPR-Cas9 technology and measured the half-life of REST in each line. As seen in *Scyl1-/-* MEFs, the half-life and steady-state level of REST were identical in both Hek293T and Hek293T-SCYL1KO cells, indicating that SCYL1 is also dispensable for REST turnover in Hek293T cells (Fig 2).

**Fig 3:**
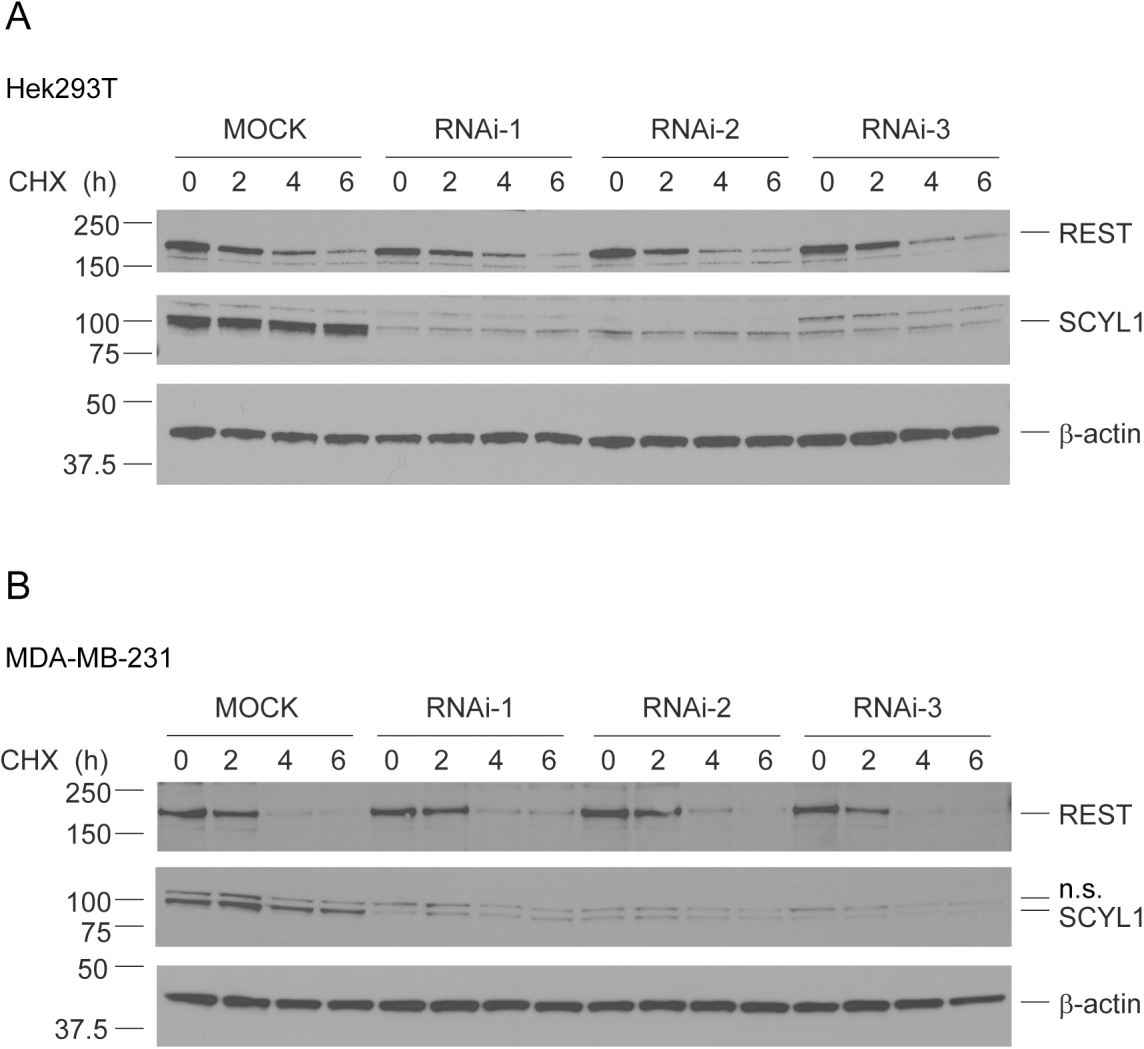
REST turnover and expression in SCYL1-depleted Hek293T cells. Hek293T (A) or MDA-MB-231 (B) cells were transfected with or without RNAi duplexes targeting *SCYL1* (RNAi-1: SCYL1HSS126245, RNAi-2: SCYL1HSS183826, RNAi-3: SCYL1HSS183827). At 48 h post- transfection, cells were treated with 10 μg/mL of CHX for the indicated time. Endogenous REST, SCYL1, and β-actin levels were determined by western blot analysis. Comparable knockdown efficiencies were achieved for all 3 RNAi duplexes.

We hypothesized that REST turnover might be differentially regulated under acute versus chronic depletion of SCYL1, which might explain the differences between our findings and those of Karlin et al. To test this, we depleted SCYL1 in Hek293T cells by using 3 distinct RNAi duplexes and measured REST half-life and turnover. Although all 3 RNAi duplexes efficiently depleted SCYL1, REST half-life and steady-state expression remained unchanged (Fig 3A). Although Karlin et al. performed most of their studies related to REST protein turnover in Hek293T cells, some of their studies were also performed in MDA-MB-231 cells. Therefore, to rule out the possibility that discrepancy between both studies is due to the use of different cell lines, we tested whether REST protein expression and turnover were differently regulated in the breast cancer cell line MDA-MB-231. We assessed the role of SCYL1 in regulating REST turnover in MDA-MB-231 by using RNAi-mediated knockdowns. Although all 3 RNAi duplexes efficiently depleted SCYL1 (Fig 3B), REST half-life and steady-state expression remained unchanged (Fig 3B). Altogether, our results indicate that SCYL1 is dispensable for the downregulation of REST.

**Fig 4:**
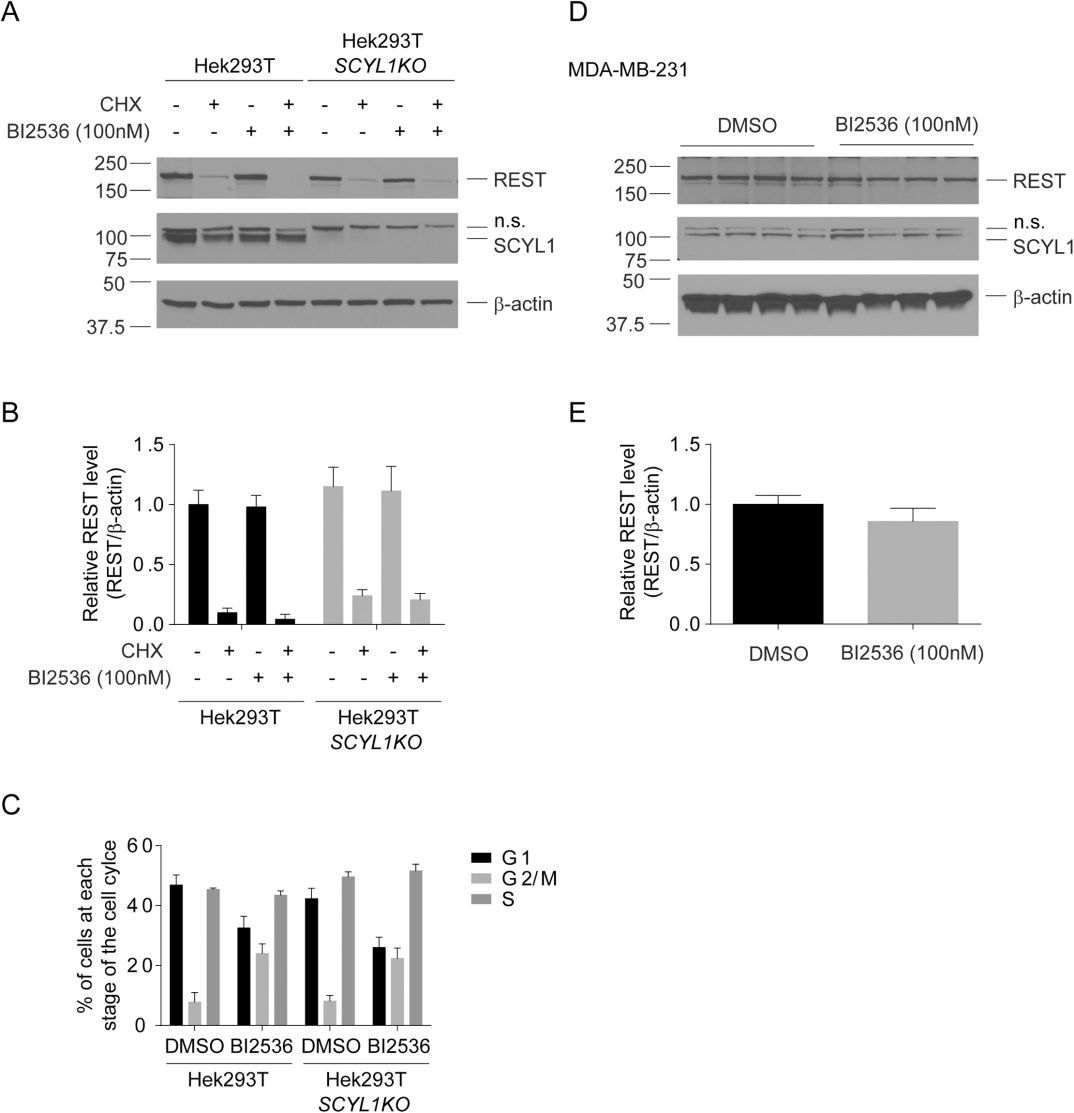
Effect of PLK1 inhibition by BI 2536 on REST protein expression and turnover. (A) Three Hek293T cell lines in which *SCYL1* was unaltered (Hek293T) and three Hek293T-SCYL1KO cell lines were seeded at a density of 250,000 cells per well of 6-well plates. The following day, the cells were treated with 10 μg/mL of CHX for 6 h in the presence or absence of absence of BI 2536 (100nM). Endogenous REST, SCYL1, and β-actin levels were determined by western blot analysis. The images are representative of 2 independent experiments performed on 3 different independently derived clones for each genotype. (B) Quantification of REST protein levels in cell lines Hek293T and Hek293T-*SCYL1KO*. The ratio of the band intensity of REST to β-actin was normalized to the ratio of untreated (time = 0 h). Data represent mean ± standard error of the mean. (C) Flow cytometric analysis of cell cycle with propidium iodide DNA staining of exponentially growing Hek293T (n = 3) and Hek293T-SCYL1KO (n = 3) cells treated for 6 h with 100nM BI 2536 or DMSO. In the presence of BI 2536, cells accumulated to 4N DNA content, indicative of G2/M arrest. Data represent mean ± standard error of the mean. (D) Four distinct cultures of MDA-MB-231 cells were incubated with DMSO or BI2 2536 (100nM) for 6 h. Endogenous REST, SCYL1, and β-actin levels were determined by western blot analysis. (E) Quantification of REST protein levels in MDA-MB-231 cells. The ratio of the band intensity of REST to β-actin was normalized to the ratio of DMSO treated cells. Data represent mean ± standard error of the mean.

Lastly, we tested whether inhibition of polo-like kinase 1 (PLK1) activity using the small molecule BI2436 affected REST protein levels and turnover in Hek293T cells as reported by Karlin et al. Surprisingly, although incubation of Hek293T or Hek293T-SCYL1KO cells with BI 2536 (100nM) for 6h induced cell cycle arrest in G2M phase (Fig 4C), consistent with previous findings [15], it failed to affect REST expression and turnover (Fig 4A and 4B). Similarly, incubation of MDA-MB-231 cells with BI 2536 (100nM) had no effect on the steady levels of REST (Fig 4D and 4E). These results indicate that PLK1 activity is also dispensable for REST turnover in both Hek293T cells and MDA-MB-231.

## Discussion

A novel oncogenic pathway regulating REST tumor suppressor turnover was recently described [7]. The STP axis, comprising SCYL1, Tex14 and PLK1, was identified in a kinome-wide RNAi screen for kinases that regulate REST turnover [7]. These findings were of particular interest to us because of our efforts in understanding the neuroprotective function of SCYL1 [9]. Thus, to validate these findings, we first analyzed REST expression and turnover in *Scyl1-/-* and *Scyl1-/-* MEFs. Unexpectedly, we found that loss of SCYL1 had no effect on REST levels and turnover. Because Karlin et al. used Hek293T cells to demonstrate that SCYL1 regulates REST turnover and because MEFs may express additional proteins that regulate REST turnover, we next targeted *SCYL1* in Hek293T cells by using the CRISPR-Cas9 technology. Again, the loss of SCYL1 did not influence REST expression and turnover. In their study, Karlin et al. used an RNAi-mediated strategy to demonstrate a role for SCYL1 in regulating REST turnover in Hek293T cells and triple-negative cancer cells. We also performed RNAi-mediated studies by using 3 distinct RNAi duplexes (SCYL1HSS126245, SCYL1HSS183826, SCYL1HSS183827, Invitrogen) in Hek293T and MDA-MB-231 cells and found that despite significantly reducing SCYL1 expression, none of the RNAi duplexes affected REST expression and turnover. Altogether, our findings clearly indicate that SCYL1 is not required for REST degradation.

The reason for the discrepancy between our results and Karlin et al. is unknown. One explanation however is that the RNAi duplexes used in their study had off-target activity. Karlin et al. initially used RNAi duplexes SCYL1HSS126244, SCYL1HSS126245, and an unspecified one to identify SCYL1 as one of the STP axis genes. HSS126244 had the most prominent effect on REST protein expression, followed by HSS126245, and the third RNAi had no effect [7]. Subsequent experiments essentially relied on SCYL1HSS126244, which was shown to affect REST protein expression levels and delay REST turnover in Hek293T cells and triple-negative breast cancer cell lines [7]. Considering that RNAi screens are notorious for producing false-positive outcomes due to gene-silencing pathways based on microRNAs [16–18], it is possible that SCYL1HSS126244, in addition to targeting SCYL1, has other target sites and thereby affects the expression of other genes that regulate REST protein expression and turnover. Functional genome-wide RNAi-based gene silencing screens have been instrumental in identifying novel cellular pathways and potential drug targets and should be continued to be used. However, potential hits from these screens should be validated by using additional robust genetic approaches such as conventional and CRISPR-Cas9–mediated gene targeting [19]. Here we used three distinct genetic strategies to directly assess whether SCYL1 is involved in regulating REST protein levels and turnover. Although REST half-life was similar in parental cell lines (Hek293T and MDA-MB-231) between the two studies, we found that depletion of SCYL1 using three distinct RNAi duplexes in MDA-MB-231 cells or Hek293T cells or by gene disruption using CRISPR-Cas9 in Hek293T cells or conventional targeting in mouse embryonic fibroblasts had no effect on REST protein level or turnover. Our findings directly challenge the mechanism put forth by Karlin et al.

Our findings also argue against a role for PLK1 activity in regulating REST protein expression and turnover and suggest that another pathway, perhaps the MAP kinase signaling pathway as shown recently [20], regulates REST turnover in Hek293T cells. Inhibition of PLK1 had no effect on REST protein expression or turnover in Hek293T and MDA-MB-231 cells. Our studies, however, do not contest the possible role of Tex14 in mediating REST turnover; the formation of an STP complex; or the possibility of SCYL1, Tex14, PLK1, and REST being aberrantly expressed in triple-negative breast cancer cells [7].
